# Repeated stereotactic radiosurgery for residual intracranial dural arteriovenous fistulas

**DOI:** 10.1007/s00701-025-06536-1

**Published:** 2025-04-29

**Authors:** Tzu-Chiang Peng, Chun-Fu Lin, Ai Seon Kuan, Hsiu-Mei Wu, Cheng-Chia Lee, Chung-Jung Lin, Huai-Che Yang

**Affiliations:** 1 Department of Neurosurgery, Neurological Institute, Taipei Veterans General Hospital, Taipei, Taiwan; 2 Department of Radiology, Taipei Veterans General Hospital, Taipei, Taiwan; 3 School of Medicine, National Yang Ming Chiao Tung University, Taipei, Taiwan

**Keywords:** Stereotactic radiosurgery, Gamma knife surgery, Dural arteriovenous fistula, Repeat, Residual, Outcome

## Abstract

**Objective:**

Stereotactic radiosurgery (SRS) is widely used for the treatment of intracranial dural arteriovenous fistulas (DAVFs); however, the outcomes of repeated SRS to deal with residual DAVFs are unclear. This study assessed the benefits and potential negative consequences of repeated SRS in patients with residual DAVFs.

**Methods:**

This retrospective study examined all patients who underwent two SRS procedures for DAVFs in a single academic medical center between January 1998 and December 2022. Information related to patient demography, DAVFs characteristics, and clinical outcomes were obtained from medical records. The objective in this study was to obtain a preliminary overview of the results of repeated SRS for DAVFs.

**Results:**

The study cohort of 19 patients included 14 patients with non-cavernous sinus (NCS) DAVFs and 5 patients with cavernous sinus (CS) DAVFs. The NCS group included 8 patients who were categorized as low-grade (Borden grade 1) and 6 as high-grade (Borden grade 2 or 3). The median follow up duration after the second session of SRS was 37 months. Symptomatic improvement was noted in 16 patients (84.2%) and total obliteration was identified in 11 patients (57.9%). No patient suffered from intracranial hemorrhage after the repeated SRS. One patient (5.3%) experienced symptomatic radiation-induced change mandating temporary course of medical treatment.

**Conclusions:**

Repeated SRS appears to be a safe and effective approach to deal with residual DAVFs, resulting in symptomatic improvement and complete radiologic obliteration.

## Introduction

Intracranial dural arteriovenous fistulas (DAVFs) are instances of abnormal blood flow between the dural artery and venous sinus or leptomeningeal vein. They account for roughly 15% of all vascular malformations of the brain [4]. Sinus thrombosis is associated with venous hypertension and the corresponding dilation of upstream capillaries, a hallmark feature of DAVFs [19]. While it is widely believed that DAVF formation is a secondary effect of inflammation, thrombosis, or trauma of the dural sinus, the exact mechanism is still debated. Another theory posits that an increase in the expression of angiogenetic factors secondary to tissue hypoperfusion leads to angiogenesis [26, 28]. The risk of bleeding is correlated with specific venous drainage patterns and specific symptoms [18, 31]. The Borden classification system is used to stratify lesions according to the type of venous drainage [2]. Asymptomatic low-grade DAVF (Borden grade 1) is associated with a lack of cortical venous drainage and benign clinical course [6], which makes it amenable to conservative management [22]. Symptomatic high-grade DAVF (Borden grade 2 or 3) generally requires direct intervention, due to an inherent risk of intracranial hemorrhage and/or neurologic deficits [7, 14]. The treatment options for DAVFs include microsurgery, embolization, and SRS [11, 12, 16], individually or in combination.

Researchers have established the feasibility and efficacy of SRS for DAVFs [13, 17], as evidenced by a complete obliteration rate of 50–80% in select patients [21, 30]. However, there has been relatively little research on the effects of repeated SRS for DAVFs. In the current study, we report on our experience in the use of SRS two times to deal with residual DAVFs.

## Methods

### Patient population

This study recruited 19 patients who underwent gamma knife radiosurgery (GKRS) twice for residual DAVFs at a single academic center between January 1998 and December 2022. This series included 14 patients with non-cavernous sinus (NCS) DAVFs and 5 patients with cavernous sinus (CS) DAVFs. The cohort included 12 males (7 females) with a median age of 53.5 years (range 25–77). The location of the fistula were as follows: Transverse-sigmoid (*n* = 9) cavernous sinus (*n* = 5), torcula (*n* = 2) and other (*n* = 3). Table 1 lists the demographic data, fistula angioarchitecture, and treatment data. Treatment included SRS alone or in combination with microsurgery and/or embolization. There were five patients receiving embolization before SRS and one among them had craniotomy earlier. All the DAVFs were identified as residual lesion before initiating the second radiosurgery. Data related to the initial presentation of symptoms was obtained from medical records. The most common symptoms were as follows: Tinnitus (*n* = 11), ocular conditions (*n* = 9), headache (*n* = 7) and muscle weakness (*n* = 2). The NCS group included 8 patients categorized as low-grade (Borden grade 1) and 6 as high-grade (Borden grade 2 or 3). Two patients had prior intracranial hemorrhage before repeated SRS. Follow-up began at repeated SRS.

**Table 1:** Characteristics of patients with DAVFs treated with SRS

Factor		Value
Male: Female		12: 7
Median age in year(range)		53.5(25–77)
Median following-up period after 2nd SRS in months(range)		37(8–193)
Location		
Cavernous		5
Transverse-sigmoid		9
Torcula		2
Superior sagittal		1
Jugular bulb		1
Clivus		1
Treatment modality		
SRS alone		14
SRS + embolization		4
SRS + embolization + surgery		1
Symptom		
Hemorrhage		2
Ocular phenomena		
Diplopia		3
Chemosis		3
Blurred vision		2
Proptosis		1
Non-ocular phenomena		
Tinnitus		11
Headache		7
Muscle weakness		2
Hearing impairment		1
Seizure		1

*DAVF* Dural arteriovenous fistula; *SRS* Stereotactic radiosurgery

### SRS procedures

Following stereotactic frame placement, patients underwent thin-slice MRI without and with intravenous contrast. Standard cerebral magnetic resonance (MR) imaging sequences were obtained for all patients. Pre-contrast sequences included fast spin echo (FSE), axial and coronal T2-weighted (3 mm sections) images, contrast-enhanced 3D volumetric (3 mm sections) MR images, and time-of-flight (TOF) MR angiography. Patients were then subjected to biplane digital subtraction angiography (DSA).

Target localization involved integrating imaging data from stereotactic non-contrast MRIs, thin-cut axial views of TOF MR angiography, and cerebral X-ray angiograms. The primary objective in this treatment was the complete occlusion of fistulous shunts. It is important to note that treatment success depended on the delineation of the treatment target, encompassing all abnormal arteriovenous shunts on the dural sinus wall. The target volume was defined relative to the dural sinus wall adjacent to which the true arteriovenous fistula occurred. Remote arterial feeders and drainage veins distal to the sinus were excluded from the treatment volume, as they were not considered part of the nidus.

### Follow-up evaluation

Patients were discharged on the day of the SRS procedure or the day after. Brain MRI was performed at intervals of 6 months after SRS. Indications of complete fistula obliteration were confirmed via catheter angiograms. Reports of patient outcomes were assessed on during regular outpatient visits. In our series, all patients received repeated SRS for infield residual DAVFs, which indicated the occurrence of residual DAVFs in the region addressed in the first SRS session.

### Statistical analysis

Patients contributed risk time from the date of completing the second SRS until the date of DAVFs complete obliteration, occurrence of any competing event, or lost to follow-up (censored), whichever was first. Cumulative function curves for DAVFs complete obliteration were plotted using competing risk analyses [9]. The competing events for DAVFs obliteration included DAVFs salvage treatments (embolization, craniotomy) and death from any cause.

## Results

### Clinical outcomes

After the second radiosurgical session, 16 of the patients (84.2%) presented sustained improvements in clinical symptoms. Four patients reported that their ailments were totally eliminated and 12 had minimal residual symptoms. Based on the initial Borden grade, symptomatic improvements were noted in all patients in the low-grade group (100%) and 3 patients in the high-grade group (50%). Three patients among high-grade group reported symptomatic deterioration or persistence (Table 2). Of note, one patient presented new-onset seizures and one patient reported exacerbated limb weakness mandating being place on the temporary course of medical treatment. Clinical outcomes are summarized in Table 3.

**Table 2:** Clinical outcomes of patient with DAVFs receiving two sessions SRS

Case	DAVF feature (NCS or CS)	Borden grade	Initial symptoms	Interval between two SRS sessions (months)	Symptomatology after first SRS	Symptomatology after second SRS	Radiologic outcomes (imaging modality)
1	NCS	Low	HA	51	Improve	Improve	CO (DSA)
2	NCS	Low	HA and tinnitus	44	Persist	Improve	CO (MRI)
3	NCS	High	HA and tinnitus	45	Improve	Persist	PR (MRI)
4	NCS	Low	Eye proptosis and bruit	55	Subside and recur	Improve	CO (MRI)
5	NCS	Low	Tinnitus	74	Subside and recur	Complete remission	CO (DSA)
6	NCS	High	Tinnitus and hemiparesis	20	Improve	Improve	CO (DSA)
7	NCS	High	HA, seizure tinnitus and ICH	30	Improve	Complete remission	CO (DSA)
8	NCS	Low	Tinnitus	35	Persist	Improve	PR (DSA)
9	NCS	High	HA and ICH	15	Deteriorate	Deteriorate	PR (MRI)
10	NCS	High	Hemiparesis	8	Persist	Improve	PR (MRI)
11	NCS	Low	HA and tinnitus	39	Improve	Improve	CO (MRI)
12	NCS	Low	HA and tinnitus	50	Subside and recur	Complete remission	CO (MRI)
13	NCS	High	Hearing impairment and tinnitus	61	Subside and recur	Persist	PR (MRI)
14	NCS	Low	Tinnitus	31	Persist	Improve	CO (DSA)
15	CS	N/A	Chemosis	51	Improve	Improve	CO (DSA)
16	CS	N/A	Chemosis	86	Improve	Improve	PR (DSA)
17	CS	N/A	Blurred vision, diplopia and tinnitus	49	Subside and recur	Complete remission	PR (MRI)
18	CS	N/A	Blurred vision, diplopia and chemosis	24	Subside and recur	Complete remission	PR (MRI)
19	CS	N/A	Diplopia	67	Subside and recur	Improve	CO (MRI)

*CS* Cavernous sinus; *NCS* Non-cavernous sinus; *HA* Headache; *ICH* Intracranial hemorrhage; *SRS* Stereotactic radiosurgery; *CO* Complete obliteration; *PR* Partial regression; *MRI* Magnetic resonance imaging; *DSA* Digital subtraction angiography; *N/A* Not applicable

**Table 3:** Summary of DAVFs clinical outcomes after second session SRS

	Complete improvement	Partial improvement	Persistence or deterioration
CS	2	3	0
NCS			
Low grade	2	6	0
High grade	1	2	3

*DAVF* Dural arteriovenous fistula; *SRS* Stereotactic radiosurgery; *CS* Cavernous sinus; *NCS* Non-cavernous sinus

### Radiologic outcomes

**Fig. 1:**
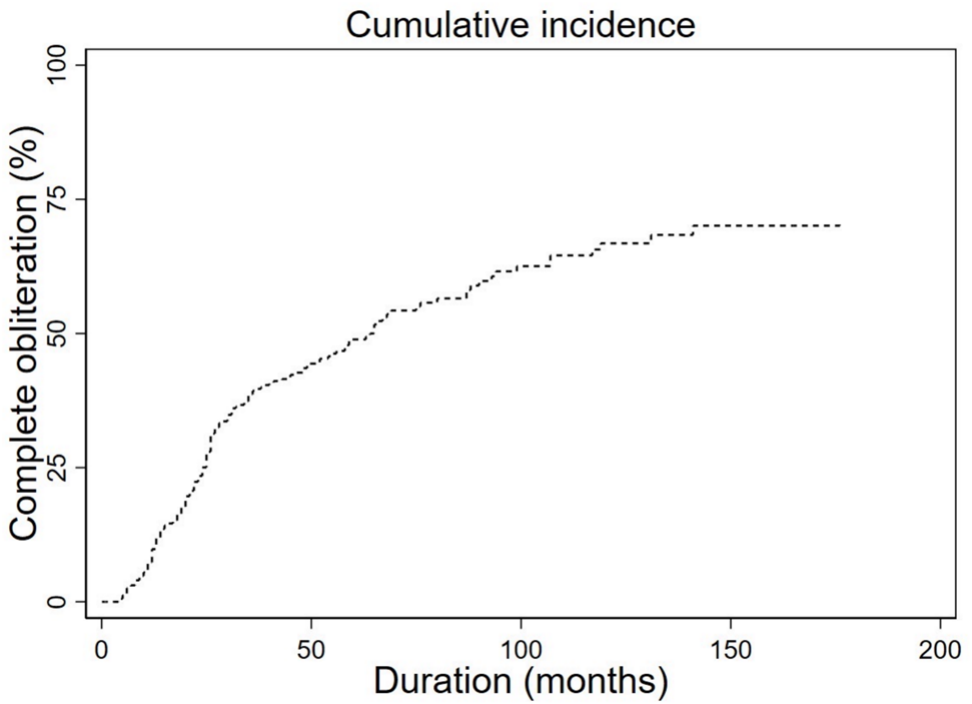
Kaplan–Meier curve indicating the time to total DAVF obliteration after the second SRS session. SRS = stereotactic radiosurgery; DAVF = dural arteriovenous fistula

Follow-up imaging studies were available for all of patients. Table 4 outlines the SRS parameters. Among the total 19 patients, 11 presented complete obliteration, 8 patients presented partial fistula occlusion, and none presented fistula progression at the time of the last angiogram, as confirmed by MRI (*n* = 11) or catheter angiogram (*n* = 8). The median interval between the second SRS session and the last imaging study was 37 months (range 8–193). The time to radiologic response is plotted as a Kaplan Meier line in Fig. 1. The overall complete obliteration rate was 57.9%.

**Table 4:** Treatment parameters of 19 patients treated with repeated SRS for DAVFs

Case	SRS session	Isocenter number	Radiation volume (cm^3^)	Max dose (Gy)	Marginal dose (Gy)	Radiation location
1	1	2	1.854	27.5	16.5	Right petrosal sinus
	2	3	1	24.62	16	Right petrosal sinus (infield)
2	1	10	8.064	36	18	Right transverse-sigmoid sinus
	2	1	1.7	20.62	17	Right transverse-sigmoid sinus (infield)
3	1	7	7.9	31.82	17.5	Left transverse-sigmoid sinus
	2	9	8.1	32.73	18	Left transverse-sigmoid sinus (infield)
4	1	8	18.9	32.69	17	Superior sagittal sinus
	2	12	13.5	30.91	17	Superior sagittal sinus (infield)
5	1	10	17.6	29.09	16	Right petrosal sinus
	2	10	7.9	28.95	16.5	Right petrosal sinus (infield)
6	1	11	9	30	18	Right transverse-sigmoid sinus
	2	16	15.1	32.7	18	Right transverse-sigmoid sinus (infield)
7	1	13	24.9	29	15.95	Left transverse-sigmoid sinus
	2	21	38.1	30.77	16	Left transverse-sigmoid sinus (infield)
8	1	17	37.5	28.95	16.5	Right transverse-sigmoid sinus
	2	14	21.2	30	16.5	Right transverse-sigmoid sinus (infield)
9	1	10	27	29.63	16	Superior sagittal sinus
	2	19	36.7	29.09	16	Superior sagittal sinus (infield)
10	1	26	59.5	29.09	16	Left transverse-sigmoid sinus
	2	14	21.7	27.27	15	Left transverse-sigmoid sinus (infield)
11	1	23	40.3	28.45	16.5	Right petrosal sinus
	2	12	18.1	29.46	16.5	Right petrosal sinus (infield)
12	1	11	2.8	27.78	17.5	Left transverse-sigmoid sinus
	2	6	0.68	30	18	Left transverse-sigmoid sinus (infield)
13	1	15	9.3	29.17	17.5	Right transverse-sigmoid sinus
	2	9	0.68	26.1	18	Right transverse-sigmoid sinus (infield)
14	1	14	16.1	30.17	17.5	Right transverse-sigmoid sinus
	2	9	12.9	28.57	16	Right transverse-sigmoid sinus (infield)
15	1	2	4.1	25	17.5	Left cavernous sinus
	2	Missing	2	24.29	17	Left cavernous sinus (infield)
16	1	5	5.3	29.09	16	Right cavernous sinus
	2	5	2.2	25	15	Right cavernous sinus (infield)
17	1	2	6.7	25	17.5	Right cavernous sinus
	2	2	2.2	25	17.5	Right cavernous sinus (infield)
18	1	5	8.6	24.29	17	Bilateral cavernous sinus
	2	2	2.1	25	17.5	Left cavernous sinus (infield)
19	1	8	3.1	24.29	17	Left cavernous sinus
	2	8	1.97	27.1	16.8	Left cavernous sinus (infield)

*SRS* Stereotactic radiosurgery; *DAVF* Dural arteriovenous fistula

Stratified by Borden grade, complete obliteration of NCS DAVFs was achieved in 7 of the 8 patients in the low-risk group and 2 of the 6 patients in the high-risk group. Two of 5 patients with residual CS DAVFs reported complete fistula remission with median interval of 33 weeks. Illustrative imaging results are summarized in Table 5.

**Table 5:** Radiologic outcomes of patient with DAVFs after second session SRS

	Complete obliteration	Partial occlusion	Progression
CS	2	3	0
NCS			
Low grade	7	1	0
High grade	2	4	0

*DAVF* Dural arteriovenous fistula; *SRS* Stereotactic radiosurgery; *CS* Cavernous sinus; *NCS* Non-cavernous sinus

## Discussion

### Outcomes of first SRS session versus follow-up SRS session

Barcia et al. [1] first reported on the use of SRS to deal with a CS fistula. Since that time, SRS has been widely adopted as an alternative approach to treating low-risk DAVFs. This technique provides a high obliteration rate in asymptomatic patients having favorable angioarchitecture without cortical venous drainage, retrograde flow, or alterations to the sinus configuration [17, 20, 21, 23, 30]. However, little is known about the outcomes when SRS is used to deal with residual DAVFs. This paper presents a series of patients who underwent a second SRS to treat residual DAVFs. In our series, patients were considered potential candidate for second session SRS as the imaging study unveiled residual fistula three years since the time point of treatment. Robert et al. promulgated a multicenter study and found more than half of obliteration occurred before three years after the radiosurgery. The imaging phenotype of our patients were carefully review and the treatment protocol was titrated case-by-case after consultation with our multidisciplinary board comprising neurosurgeons and radiation physicists. As the patients were considered proper candidates of second session radiosurgery, repeated SRS for residual fistula was suggested. Other treatment modalities comprising embolization and surgery were also well acknowledged by the patients, and the risk-to-benefit profiles of interventions were appraised by them.

**Fig. 2:**
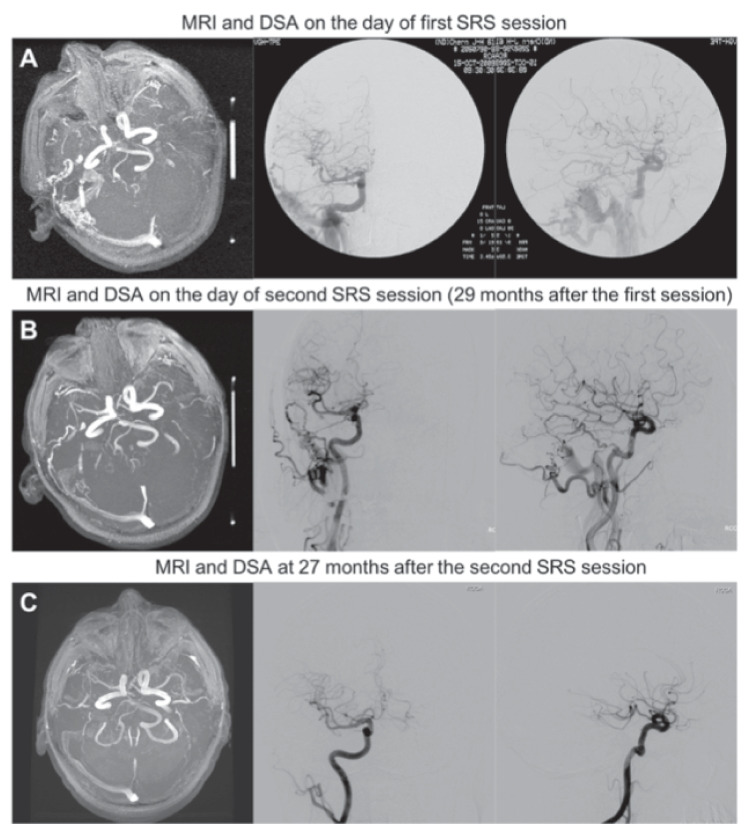
Illustrative case of patient who underwent SRS 2 times for NCS DAVF: Images from 62 years old man who presented at the hospital due to right-side tinnitus: A) Imaging results revealed early opacification of right-side sigmoid sinus supplied by the occipital artery, middle meningeal artery of external carotid artery, and meningo-hypophyseal trunk of internal carotid artery. The right-side transverse-sigmoid DAVF was identified as Borden grade 1, prompting SRS intervention; B) Residual DAVF detected at 29 months after the first SRS session, prompting a second (infield) SRS session; C) Total regression of DAVF (with corresponding resolution of symptoms) at 27 months after the second SRS session. MRI = magnetic resonance imaging; DSA = digital subtraction angiography; SRS = stereotactic radiosurgery; DAVF = dural arteriovenous fistula

In the current study, the radiology-confirmed rate of complete obliteration after second session SRS was 57.9% (11/19), which is consistent with the results in previous studies (Table 6). An illustrative case is depicted in Fig. 2.

**Table 6:** Review of the literature pertaining to radiosurgery for DAVFs

Study	Cases (n)	Mean follow-up period (months)	Complete obliteration rate
Pan et al. 2002	NCS fistula	19	
	Low grade: 10		70%
	High grade: 11		44%
Koebbe et al. 2005	CS fistula: 5	46	60%
	NCS fistula		
	Low grade: 6		83%
	High grade: 3		100%
Söderman et al. 2006	NCS fistula	24	
	Low grade: 16		75%
	High grade: 36		44%
Yang et al. 2010	CS fistula: 17	45	94%
	NCS fistula		
	Low grade: 16		63%
	High grade: 11		55%
Park 2017	CS fistula: 18	33	83%
	NCS fistula		
	Low grade: 11		79%
	High grade: 19		73%
Hung et al. 2020	CC fistula: 20	26	85%
	NCS fistula: 111	34	59%

*DAVF* Dural arteriovenous fistula; *NCS* Non-cavernous sinus; *CS* Cavernous sinus

In the current series, favorable outcomes (complete obliteration and no intracranial hemorrhage) were observed in 11 patients of the 19 patients. The likelihood of a complete obliteration was higher among patients identified as Borden grade 1 NCS DAVFs (87.5%) than among patients identified as Borden grade 2 or 3 (33.3%). The likelihood of a symptomatic improvement was also higher among patients identified as Borden grade 1 (100%) than among patients identified as Borden grade 2 or 3 (50%). These results are congruent with those in the literature that presence of cortical venous drainage conferred more malignant clinical course and lower complete obliteration rate after SRS [1, 20, 21].

**Fig. 3:**
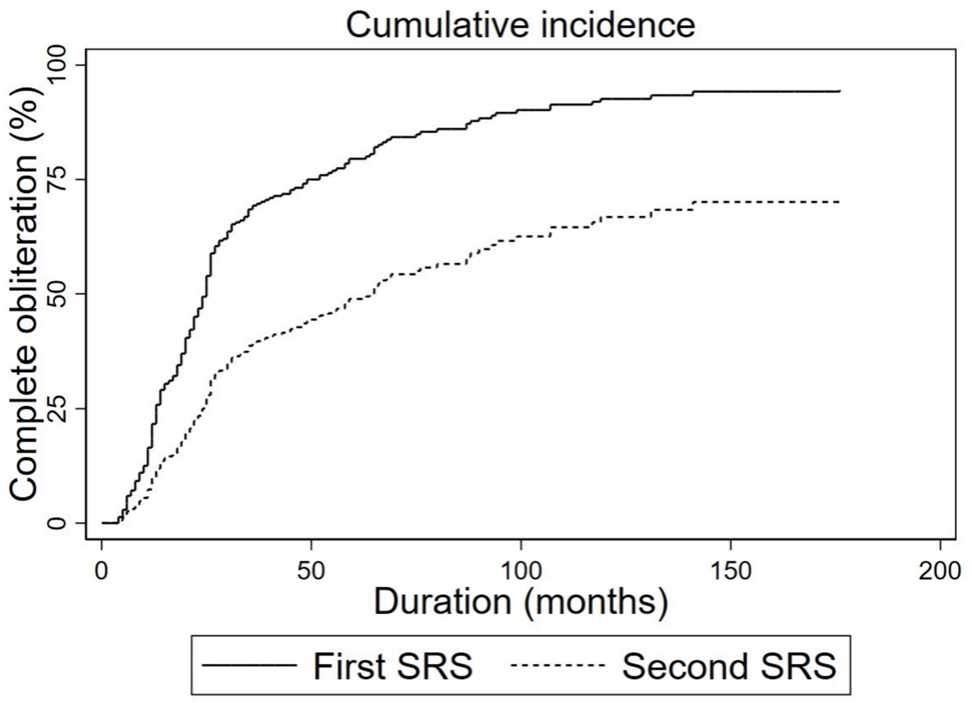
Kaplan–Meier curve indicating the time to complete obliteration rates among patients who underwent one SRS session and those who underwent two sessions for residual DAVFs. SRS = stereotactic radiosurgery; DAVF = dural arteriovenous fistula

Figure 3 denoted the cumulative rate of complete obliteration of single session SRS during the same period from January 1998 to December 2022 in our hospital. The cohort included 331 subjects harboring 336 DAVFs. Five of the 331 patients had two separate DAVFs that each received one SRS session. The fiver-year complete obliteration rate was calculated 57.4%, noting that this corresponds to the complete obliteration rate for low-risk DAVFs in previous studies (50–80%). We speculate that the relatively poor response to the second SRS session can be attributed to confounding factor that the proportion of patients classified as Borden grade 2 or 3 was higher in the group that underwent a second SRS session (31.6%; 6/19) than in the group that underwent only one session (23.2%; 78/336). Numerous publications had demonstrated higher Borden grade conferred lower obliteration rate and the nuance in baseline angiographic characters could have influence on final outcomes.

### Repeated SRS is a safe procedure with low complication rates

Despite the widespread use of SRS for the treatment of DAVFs, researchers have reported a number of SRS-related complications [21, 30], including radiation-induced brain injury, cranial nerve deficit, or intracranial hemorrhage. Soderman et al. stated that the incidence of intracranial hemorrhage after SRS was 2.5% [24]. Yang et al. reported radiation-induced brain edema in 5.5% of patients and new-onset cranial nerve deficits in 0.9% of patients [30].

In the current series, one patient presented symptomatic radiation-induced change after the second SRS sessions. The overall incidence of complications associated with the second SRS session was 5.3% (1/19). This patient was initially diagnosed with Borden grade 3 DAVF at right side transverse-sigmoid sinus (case 7). The patient was treated two sessions of infield radiosurgery with the interval of 30 months. Calculated from the time of last SRS, complete obliteration occurred 30 months later with corresponding complete remission of initial symptom. However, progressive left side homonymous hemianopia developed during the subsequent years. The follow-up brain MRI exhibited evidence of right side occipital lobe perilesional edema suggestive of untoward adverse effect of radiation. The patient underwent continuous clinical evaluation and was placed on a temporary course of steroid. The hemianopia stabilized and demonstrated no progression over the next 9 years of follow up.

Intracranial hemorrhage was another clinically relevant issue portending devastating morbidity after SRS. Pertinent study by Rober et al. concluded patients have similar rates of hemorrhage, before and after radiosurgery until obliteration is achieved with overall annual risk of 0.84% [25]. In the current study, none had hemorrhage during the latent period after the second session SRS.

### Advantages of SRS for the treatment of DAVFs

Patients with complex DAVFs (e.g., multiple arterial feeder, diffuse venous drainage, or proximity to eloquent areas) may face an elevated risk of complications during surgery and/or embolization [5]. It is important to consider that once the sinus drainage is embolized or resected, unpredictable changes in hemodynamic flow can have catastrophic results [15, 16, 27]. Thus, it is important to consider a multidisciplinary approach to the treatment of complex DAVFs. From a theoretical standpoint, embolization can have immediate effects, while SRS poses the risk of delayed fistula closure. This has prompted a number of researchers to promote SRS in conjunction with embolization [10, 29]. A systemic review by Chen et al. (729 patients harboring 743 DAVFs) demonstrated that SRS provided favorable outcomes and an acceptable complication profile when treating DAVFs (with or without CVD) [3]. The author recommended that SRS is a viable non-invasive approach to treating DAVFs, providing durable local control and positive outcomes. In the current study, repeated SRS was shown to be an effective and reasonable salvage therapy for residual DAVFs with low complication risk.

### Study limitations

As with any retrospective study, the current series was subject to inherent bias, and the rarity of DAVFs limited the statistical power of our findings. It is important to consider that 57.9% of the patients (11/19) were unwilling to proceed with a catheter angiogram after receiving MRI confirmation of complete obliteration. However, growing evidence demonstrated MRI was a highly reliable technique in the screening and surveillance of DAVFs with excellent accuracy [8]. Another issue is that the study included patient treated at different periods. Over an extended time frame, various Gamma knife models or neuroimaging technologies were introduced. It is also believed that steady accumulation of experience and expanded knowledge can lead to more favorable outcomes in the later period to deal with these lesions. Further large-scale, prospective and randomized studies are demanded to support our findings and broaden our appreciation of this subject.

## Conclusions

Most of the patients who underwent a second SRS session benefited from sustained clinical improvements. The likelihood of favorable outcomes after the second SRS session depended on the underlying risk (Borden grade). The likelihood of complete fistula obliteration was higher among patients designated as Borden grade 1 than among those designated as grade 2 or 3. The overall complication rate among patients who underwent a second SRS session was low. The preliminary results of the current study lend credence to second session SRS as a salvage therapy for DAVFs.

## Data Availability

No datasets were generated or analysed during the current study.
